# Combined immunodeficiency develops with age in Immunodeficiency-centromeric instability-facial anomalies syndrome 2 (ICF2)

**DOI:** 10.1186/s13023-014-0116-6

**Published:** 2014-10-21

**Authors:** Horst von Bernuth, Ethiraj Ravindran, Hang Du, Sebastian Fröhler, Karoline Strehl, Nadine Krämer, Lina Issa-Jahns, Borko Amulic, Olaf Ninnemann, Mei-Sheng Xiao, Katharina Eirich, Uwe Kölsch, Kathrin Hauptmann, Rainer John, Detlev Schindler, Volker Wahn, Wei Chen, Angela M Kaindl

**Affiliations:** Pediatric Pneumology and Immunology, Charité - Universitätsmedizin Berlin, Augustenburger Platz 1, 13353 Berlin, Germany; Labor Berlin Charité Vivantes GmbH, Department of Immunology, Berlin, Germany; Institute of Cell Biology and Neurobiology, Charité – Universitätsmedizin Berlin, Augustenburger Platz 1, 13353 Berlin, Germany; Pediatric Neurology, Charité - Universitätsmedizin Berlin, Augustenburger Platz 1, 13353 Berlin, Germany; Berlin Institute for Medical Systems Biology, Max-Delbrueck-Center for Molecular Medicine, Robert-Rössle-Str. 10, 13092 Berlin, Germany; Max Planck Institute for Infection Biology, Berlin, Germany; Institute for Human Genetics, Biozentrum, Universität Würzburg, Würzburg, Germany; Institute for Pathology, Charité – Universitätsmedizin Berlin, Berlin, Germany

**Keywords:** ZBTB24, ICF2, Immunodeficiency, Microcephaly, Intellectual disability, Centromeric instability, Facial anomalies, Granulomas

## Abstract

The autosomal recessive immunodeficiency-centromeric instability-facial anomalies syndrome (ICF) is characterized by immunodeficiency, developmental delay, and facial anomalies. ICF2, caused by biallelic *ZBTB24* gene mutations, is acknowledged primarily as an isolated B-cell defect. Here, we extend the phenotype spectrum by describing, in particular, for the first time the development of a combined immune defect throughout the disease course as well as putative autoimmune phenomena such as granulomatous hepatitis and nephritis. We also demonstrate impaired cell-proliferation and increased cell death of immune and non-immune cells as well as data suggesting a chromosome separation defect in addition to the known chromosome condensation defect.

**Electronic supplementary material:**

The online version of this article (doi:10.1186/s13023-014-0116-6) contains supplementary material, which is available to authorized users.

## Letter to the editor

The autosomal recessive immunodeficiency-centromeric instability-facial anomalies (ICF) syndrome is characterized by immunodeficiency, intellectual deficit, and facial dysmorphism [1]. ICF 1 and 2 are caused by biallelic mutations in the DNA methyltransferase 3B gene *DNMT3B* (MIM*602900, [2,3]) and in the zinc-finger-and BTB-domain containing 24 gene *ZBTB24* (MIM*614064, [4]), respectively. For ICF2, 16 patients from 13 families have been reported (Additional file 1: Table S1) [4-13]. ICF is considered primarily as a humoral immunodeficiency disease; however, this does not explain the high rate of opportunistic infections. Recently, an additional intrinsic T-cell deficiency in ICF has been discussed and a lymphocyte proliferation defect described in individual patients with ICF1 and ICF2 [5,8,9]. Mechanisms underlying the neurological phenotype of ICF remain to be elucidated. Here, we report the development of a combined immunodeficiency in a patient with ICF2 with age and demonstrate pathomechanisms that may contribute to the immunological and non-immunological phenotype.

**Figure 1:**
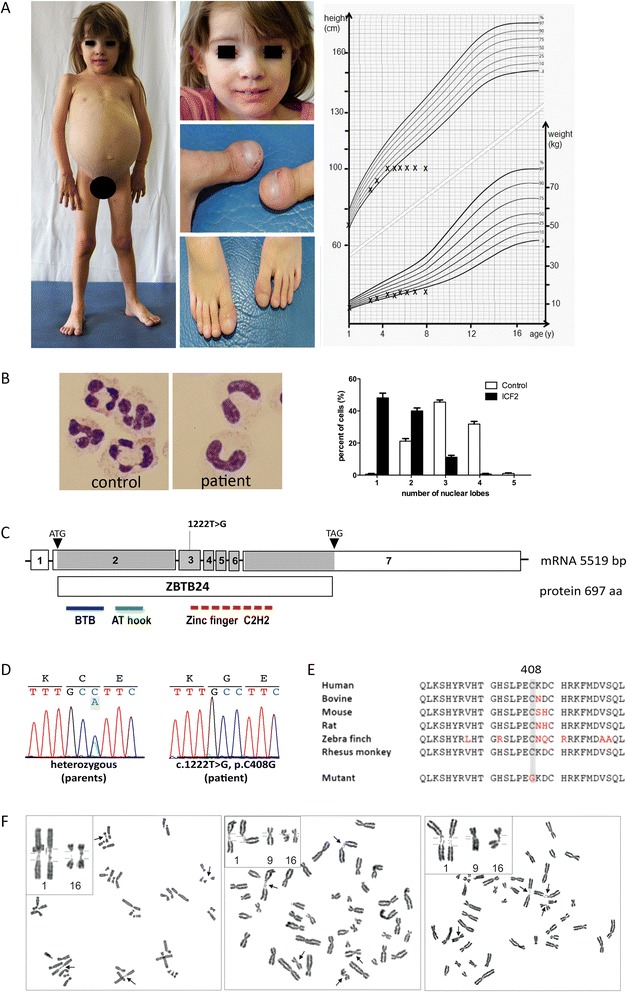
**Phenotype and genotype of index patient with** ***ZBTB24*** **mutation. (A)** Clinical signs at 8 years-of-age: protruding abdomen due to organomegaly in the otherwise underweight girl of short statue, facial dysmorphism (hypertelorism, epicanthal folds, flat nasal bridge, hypertelorism, slight ptosis, prominent forehead). Large teeth result from a fusion of first molar with the incisors. Fingers and toes showed clubbing. Failure to thrive evident in a percentile height-weight-curve. **(B)** Pseudo-Pelger-Huët anomaly of neutrophils (Diff-Quick staining, 100x, n = 400 cells, Student’s t-test, p < 0.0001). **(C)** Site of homozygous *ZBTB24* mutation c.1222 T > G (protein domains: BTB, bric-a-bric, tramtrack, broad complex domain; AT hook, DNA-binding domain with a preference for A/T rich regions, Zinc finger C2H2). **(D)** Electropherogram traces in patient and heterozygous parents (NM_014797) indicating mutation confirmed by Sanger sequencing. Unaltered *ZBTB24* mRNA levels and product size is depicted in Additional file 8: Figure S2. **(E)** Highly conserved amino acids affected by the inherited homozygous mutation (p.C408G). **(F)** Spontaneous undercondensation of constitutive heterochromatin of chromosomes 1q, 16q, and (to a lesser extent) 9q.

The index patient was born hypotrophic at term without complications as the first child of non-consanguineous healthy, Caucasian parents of German descent after an uneventful pregnancy. She showed multiple facial anomalies, clubbing of fingers and toes, and fused teeth (Figure 1A). Language and motor development appeared initially normal, but intellectual disability became apparent by the second year of life. Her brain morphology was normal on MRI at 4 years-of-age, apart from a pineal cyst. Growth stagnated at 4.5 years-of-age with height, weight, and head circumference of 101 cm (-4.79 SD), 15 kg (-2.51 SD), and 50 cm (-1.2 SD) at 9 years-of-age (Figure 1A). Bone age was delayed by 4 years at 8 years-of-age, and growth hormone levels were undetectable but could be stimulated.

Signs suggestive of an immune defect were recurrent infections of the upper airways beginning at 9 months, a pneumonia at 2.5 years-of-age (*Enterobacter cloacae*), recurrent and protracted diarrhoea (enteropathogenic *E. coli*), and a prolonged skin infection (*Streptococcus pyogenes*). At 3 years-of-age, a ‘common variable immunodeficiency (CVID)’ was diagnosed based on the global reduction of immunoglobulins and the lack of antibodies against recall antigens despite regular vaccination (Additional file 2: Table S2), subcutaneous IgG substitution was started, and the patient subsequently remained free of invasive infections. Further analysis revealed microcytic hypochrome anemia with anisocytosis, normal global T-/B-cell counts, intermittent neutropenia, and Pseudo-Pelger-Huët anomaly of neutrophilic granulocytes suggestive of a terminal neutrophil differentiation defect (Additional file 3: Table S3, Figure 1B). The patient (blood group 0) lacked isohemagglutinins against blood group substances A and B. Starting at 3.5 years-of-age, CD8+ T-cells were elevated and CD4+ T-cells dropped, leading to a profoundly reduced CD4/CD8 ratio (Additional file 3: Table S3). In parallel to the CD8+ T-cell expansion, relative numbers of CD4 + CD45RA + naïve T-cells declined, resulting in a relative CD4 + CD45R0+ T-cell increase (Additional file 3: Table S3). The TCRVβ-repertoire was normal, and bone marrow analysis excluded myelodysplasia. Lymphocyte proliferation was strongly reduced upon stimulation with mitogens, CD3-directed antibody, and tetanus toxoid despite appropriate tetanus vaccination (Additional file 4: Table S4). Initially normal NK-cell numbers declined gradually, and diminished NK-cell mediated lysis could be restored only partly through IL-2 addition (Additional file 3: Table S3, Additional file 5: Table S5).

Massive hepatosplenomegaly developed by 3 years that progressed to liver cirrhosis by 9 years-of-age. Repeated liver biopsies at 4.5 and 8.5 years-of-age revealed chronically active interface hepatitis with periportal lymphoid infiltrates and fibrosis (Additional file 6: Figure S1); no infection with hepatotopic viridae (cytomegalovirus, Epstein Barr virus, herpes viridae (HSV1, HSV2, HHV6, HHV7), hepatitis viridae A-E, adenovirus, enterovirus, parvovirus B19, hantavirus, and human polyoma virus BK-virus) or mycobacteriae was detected (Additional file 7: Table S6). Compensated renal insufficiency at 4.5 years-of-age prompted a kidney biopsy that revealed interstitial granulomatous nephritis (Additional file 6: Figure S1), but no evidence of mycobacterial infection through PCR, staining procedure, and direct culture. A four-week course of immunosuppressive treatment with corticosteroids and azathioprine was not successful in normalizing liver enzymes or kidney function. The increase of CD8+ T-cells is likely an autoimmune phenomenon non-responsive to standard immunosuppressive treatment; however, an ongoing, non-identified chronic infection cannot be ruled out. The increase of IgA, IgM, and IgG later in life can be an effect secondary to progressive liver cirrhosis. The patient is still under IgG-substitution, and protein-electrophoresis revealed no indication of an increase of mono- or oligoclonal immunoglobulins (data not shown).

**Figure 2:**
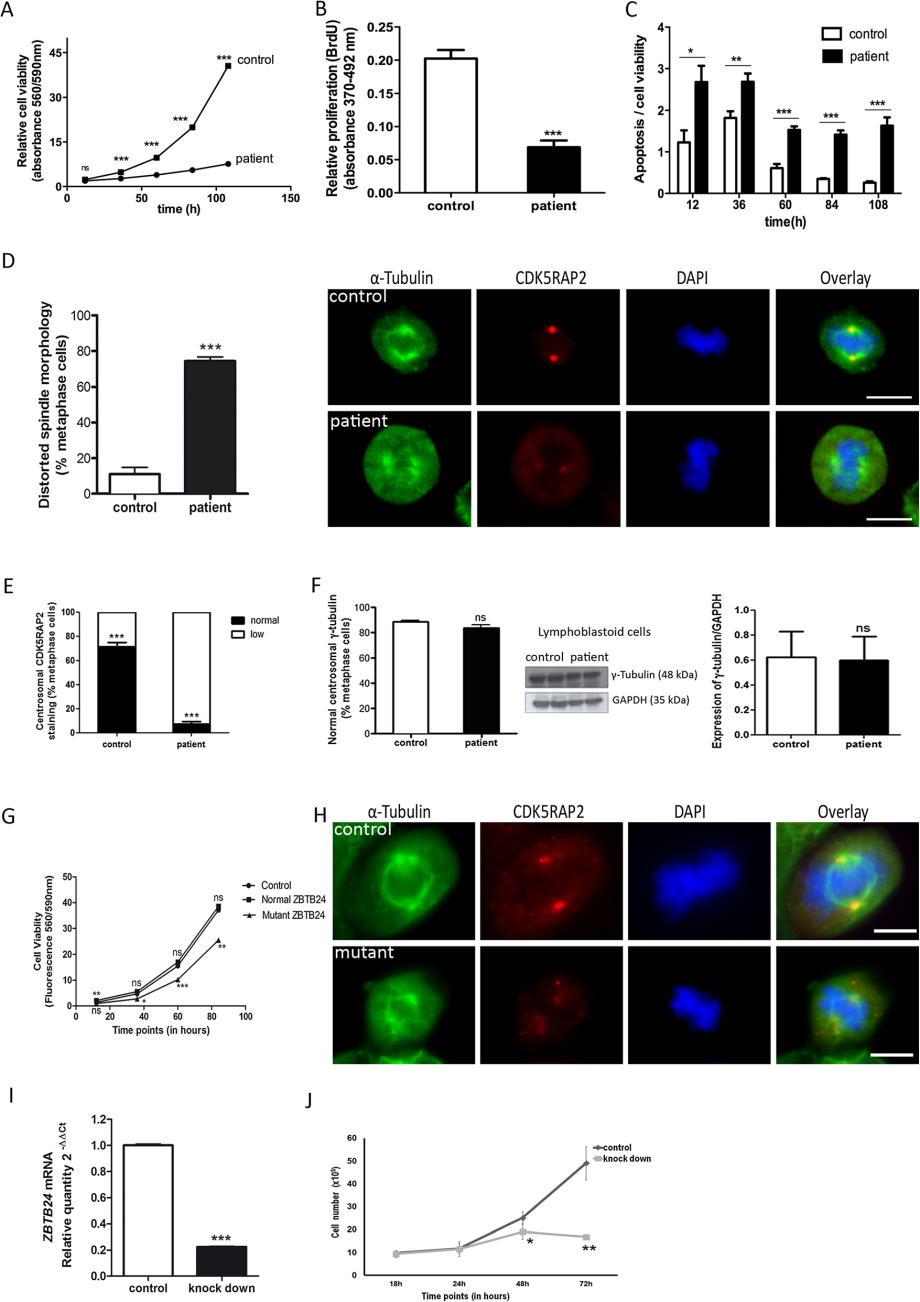
**Cellular defects in patient fibroblasts and lymphoblastoid cells, reproduced in HEK cells through siRNA and expression of mutant ZBTB24. (A)** Reduced cell viability (n = 8 per time period, Student’s t-test), **(B)** reduced proliferation (n = 8, 36 h after plating, Student’s t-test), and **(C)** increased apoptosis of *ZBTB24* mutant fibroblasts (activated caspase 3/7 per cell viability; n = 8, Student’s t-test). **(D)** Abnormal spindle (α-tubulin) formation with increase of slightly broader, unfocused microtubules poles (n = 100 metaphase LCLs, Student’s t-test, view Additional file 9: Figures S3 for further images throughout the cell cycle and specifically in metaphase cells) and **(E, F)** strongly reduced fluorescence signals of centrosomal marker CDK5RAP2 (but not γ-tubulin) in patient lyphoblastoid cells (n = 105 metaphase cells, Student’s t-test); total γ-tubulin levels were unaltered (n = 3, Student’s t-test). Scale bar 5 μm. View Additional file 10: Figures S4 for further images throughout the cell cycle and specifically in metaphase cells. **(G)** Reduced cell viability (n = 8 per time period, One-way ANOVA) and **(H)** mitotic spindles defect in HEK cells expressing mutant (c.1222 T > G) *ZBTB24*. Abnormal spindle formation with slightly broader, unfocused microtubule poles in mutant cells. Scale bar 5 μm. View Additional file 11: Figure S5 for further images throughout the cell cycle, qPCR and Western blot results. **(I)** *ZBTB24* mRNA downregulation in HEK through siRNA (qPCR, Student’s t-test) causes **(J)** reduced cell culture growth (n = 3 per group per time period; Student’s t-test). ns = not significant, *p < 0.05, **p < 0.01, ***p < 0.001.

By whole exome sequencing, we identified the homozygous missense mutation c.1222 T > G of the *ZBTB24* gene (NM_014797) in the index patient inherited from the healthy parents (Figure 1C). This previously described mutation alters evolutionarily conserved amino acids in a highly conserved zinc finger domain (p.C408G; Figure 1D,E, Additional file 1: Table S1). Patients with the c.1222 T > G mutation show a variable phenotype, arguing against a clear genotype-phenotype correlation and for a residual activity of mutant ZBTB24. In line with this, *ZBTB24* mRNA levels did not differ significantly between patient and control (Additional file 8: Figure S2). Chromosome metaphase preparations revealed increased rates of undercondensated juxta-centromeric chromosomes 1q, 16q, and less frequent of 9q regions, characteristic for ICF2 (Figure 1F). This further increased upon exposure of cultures to 5-azacytidine (DNA methylation interfering agent), eventually resulting in chromosome instability (data not shown). Because ZBTB24 not only impacts on immune cells, we examined patient fibroblasts and detected significantly reduced proliferation, increased apoptosis and discrete spindle defects (broader and unfocused microtubule poles; Figure 2A-D, Additional file 9: Figure S3). In mutant cells, centrosomal CDK5RAP2 was strongly reduced, while centrosomal y-tubulin staining and total y-tubulin levels were normal (Figure 2E,F, Additional file 10: Figure S4). The mechanisms underlying the reduction in CDK5RAP2, which is associated with stem cell proliferation and microcephaly with intellectual deficit [14], may be involved in the pathogenesis of the neurological phenotype of ICF.

We further mimicked the situation in the patient through *ZBTB24* siRNA knockdown experiments in HEK293 cells and over-expressed mutant and wild-type ZBTB24 in these cells (Figure 2G-J). Cell culture growth was significantly reduced when mutant, but not wild-type, ZBTB24 was expressed, and both discrete spindle defects and an abnormal centrosomal CDK5RAP2 staining were observed (Figure 2G,H, Additional file 11: Figure S5). Down-regulation of ZBTB24 through siRNA similarly reduced cell culture growth (Figure 2 I,J).

The novelty of our report lies in the description of the development of a combined immunodeficiency (CID) with age in ICF2, a feature that may be missed if immunological work-up is only performed once at a young age. We also highlight findings consistent with autoimmune phenomena (hepatitis, nephritis), which are commonly seen in CID but not acknowledged for ICF [15]. Finally, we report for the first time a defect in cell survival and proliferation in immune and non-immune cells. This may constitute a disease mechanism common for both the non-immunological and immunological features of ICF2, the latter presenting as CID. The clinical course of the index patient and of previously reported patients calls for consideration of early stem cell transplantation as an option in patients with ICF.

Please see Additional file 12: Materials and Methods for details on materials and methods.

## Consent statement

Written informed consent was obtained from the patient’s legal guardian for publication of this case report and any accompanying images. A copy of the written consent is available for review by the Editor-in-Chief of this journal.

## Additional files

Additional file 1: Table S1 Genotype and phenotype of all published ICF2 patients. Additional file 2: Table S2 Immunoglobulin levels and antibody titers. Additional file 3: Table S3 Blood counts and lymphocyte subpopulations. Additional file 4: Table S4 Lymphocyte proliferation assays. Additional file 5: Table S5 NK-cell cytotoxicity assay. Percentages of lysed K562 cells at different effector:target ratios with and without supplementation with IL-2 at the age of 4 years are shown. Additional file 6: Figure S1 Histological biopsy results. (A) Liver biopsy at the age of 4,5 years. Portal lymphocytic infiltrates and interface hepatitis. (B) Liver biopsy at the age of 8,5 years. Porto-portal bridging. (C) Kidney biopsy at the age of 4,5 years. Normal glomerula. (D) Kidney biopsy at the age of 4,5 years showed multifocal inflammatory infiltrates in cortex and medulla of the tubulo-interstitium and a multinuclear giant cell. Additional file 7: Table S6 Pathogens excluded to cause hepatitis. Additional file 8: Figure S2 ZBT24 mRNA levels in controls and fibroblasts. (A) RT-PCR of *ZBTB24* in fibroblasts of controls and the patient. (B) Quantitative RT-PCR of *ZBTB24* in fibroblasts of a control cell line and the patient. Additional file 9: Figure S3 Mitotic spindles defect in *ZBTB24* mutant patient cells. Subcellular localization of the spindle marker α-tubulin (green) and the centrosome marker CDK5RAP2 (red) of immortalized lymphocytes of (A) control and (B) ICF2 patient throughout the cell cycle. DNA was stained with DAPI (blue). Patient cells have abnormal spindle formation with an increase of slightly broader, unfocused microtubules poles. The fluorescence signals of the centrosomal marker CDK5RAP2 are strongly reduced in patient cells when compared to control cells. Immunofluorescence, scale bar 5 μm. Additional file 10: Figure S4 Abnormal CDK5RAP2 and normal γ-tubulin staining of centrosomes in *ZBTB24* mutant patient cells. (A) Subcellular localization of the centrosome marker CDK5RAP2 (red) in the metaphase of immortalized lymphocytes of control and ICF2 patient. DNA was stained with DAPI (blue). Centrosomal CDK5RAP2 is strongly reduced in *ZBTB24* mutant lymphocytes when compared to controls. (B) Subcellular localization of the centrosome marker γ-tubulin (green) in the metaphase of immortalized lymphocytes of control and ICF2 patient. DNA was stained with DAPI (blue). No strong difference between the γ-tubulin immunostaining of control and ICF2 patient Immunofluorescence, scale bar 5 μm. Additional file 11: Figure S5 Mimicking ICF2 in HEK cells. (A) *ZBTB24* mRNA levels and in mock-transfected HEK cells (“control”), HEK cells transfected with HA-tagged normal *ZBTB24* or mutant c.1222 T > G *ZBTB24*, assessed by qPCR. (B) Western-Blot of ZBTB24 of protein extracts from similarly transfected HEK cells using antibodies directed against HA-tag and against ZBTB24 (reference proteins GAPDH and actin). (C) Subcellular localization of the centrosome marker γ-tubulin (green) and the centrosome marker CDK5RAP2 (red) throughout the cell cycle in mock-transfected HEK cells (“control”) and HEK cells transfected with mutant c.1222 T > G *ZBTB24.*
Additional file 12
**Materials and Methods.**

## Abbreviations

CID: Combined immunodeficiency
*DNMT3B*: DNA methyltransferase 3B gene
ICF: Immunodeficiency-centromeric instability-facial anomalies syndrome
SD: Standard deviation
*ZBTB24*: Zinc-finger-and BTB-domain containing 24 gene
