# Supplementary material for: Combined immunodeficiency develops with age in Immunodeficiency-centromeric instability-facial anomalies syndrome 2 (ICF2)
Source: Orphanet J Rare Dis. 2014 Oct 21;9:116. doi: 10.1186/s13023-014-0116-6 (PMC4230835; doi:10.1186/s13023-014-0116-6)
Supplement: Additional file 1: Table S1 — Genotype and phenotype of all published ICF2 patients. [file 13023_2014_116_MOESM1_ESM.docx]

| **Additional file 1: Table S1. ICF2 phenotypes and genotypes** | | | | | | | | | | | | | | |
| --- | --- | --- | --- | --- | --- | --- | --- | --- | --- | --- | --- | --- | --- | --- |
| **Mutation** | **Protein** | **Ethnicity** | **Sex** | **Age** | **Arthropo-metric data** | **Facial anomalies** | **Motor develop-ment** | **Intellec-tual develop- ment** | **Infections** | **B Cells** | **T Cells** | **NK cells** | **Miscell-aneous** | **Ref.** |
| c.47C>G | p.S16* | Scottish | f | died at 13 y | birth weight 1900g  weight <P3  height < P3  HC < P3 | triangular face  upturned nose  flattened nasal bridge  frontal bossing  sparse dry hair | Normal | talked three words at 25 months | Pneumocystis jiroveci  pneumonia | **IgG 1.5 g/l**  (**5 – 13 g/l)**  **IgM 0,22 g/l**  **(0,36 – 1,92 g/l)**  **IgA 0,07 g/l (0,26 – 1,47 g/l)** |  |  |  | 1,2,6,7 |
| c.501dup | p.V168S erfsX28 | Turkish | m | 13 y |  | Present (not specified) |  | delayed |  | “**agamma-globulinemia**” |  |  |  | 1,6 |
| c.396_397delTA | p.H132Q fs*19 | Lebanese | m | 13 y | HC P5-15, height >P5, | round face  high arched palate  small chin retrognathism  everted lower lips | walked at 2 ½ y | talked at 4 y |  | “normal B cell count”  **∅ CD19+CD27+ memory B cells**  IgG 7,69 g/l  (7 – 16 g/l)  **IgM 0,185 g/l**  **(0,4 – 2,3 g/l)**  IgA 1,88 g/l  (0,7 – 4,3 g/l)  IgG1 6,09 g/l  (4,9 – 11,4 g/l)  IgG2 1,8 g/l  (1,5 – 6,4 g/l)  IgG3 1,6 g/l  (0,2 – 1,1 g/l)  IgG4 < 0,04 g/l  (0,08 – 1,4 g/l) | “normal T cell count”  **↑ CD4+CD45RA+ ↓ CD4+CD45RO+** | “normal NK cell count” |  | 4,6 |
| c.396_397delTA | p.H132Q fs*19 | Lebanese | m | 12 y | HC P5-15, height >P5, | round face  high arched palate  small chin  retrognathism  everted lower lips | walked at 2 ½ y | talked at 4 y |  | “normal B cell count”  **∅ CD19+CD27+ memory B cells**  **IgG 5,33 g/l**  **(8 – 18 g/l)**  **IgM 0,221 g/l**  **(0,75 – 2,5 g/l)**  IgA 1,76 g/l  (0,8 – 4 g/l)  IgG1 4,67 g/l  (4 – 11,5 g/l)  IgG2 1,08 g/l  (0,98 – 4,8 g/l)  IgG3 1,94 g/l  (0,15 – 1,49 g/l)  IgG4 < 0,04 g/l  (0 - 2,1 g/l) | “normal T cell count”  **↑ CD4+CD45RA+ ↓ CD4+CD45RO+** | “normal NK cell count” |  | 4,6 |
| c.396_397delTA | p.H132Q fs*19 | Lebanese | m | 7 y | HC P5-15, height >P5, | round face  high arched palate  small chin  retrognathism  everted lower lips | walked at 2 ½ y | talked at 4 y |  | “normal B cell count”  **∅ CD19+CD27+ memory B cells**  **IgG 7,69 g/l**  **(8 – 18 g/l)**  **IgM 0,185 g/l**  **(0,75 – 2,5 g/l)**  IgA 1,88 g/l  (0,8 – 4 g/l)  IgG1 6,09 g/l  (4 – 11,5 g/l)  IgG2 1,8 g/l  (0,98 – 4,8 g/l)  IgG3 1,6 g/l  (0,15 – 1,49 g/l)  IgG4 < 0,04 g/l  (0 – 2,1 g/l) | “normal T cell count”  **↑ CD4+CD45RA+ ↓ CD4+CD45RO+** | “normal NK cell count” |  | 4,6 |
| c.759C>G | p.T253* | Turkish | m |  | normal growth | hypertelorism | normal | delay of speech |  | “normal B cell count”  **IgG 1,7 gl/l**  **IgM 0,1 g/l**  **IgA 0,07 g/l** | “normal T cell count”  “normal CD4/CD8 ratio”  “normal CD4+CD45RA+/ CD4+CD45RO+ ratio”  “normal mitogen-  (PHA, SEB-) and antigen-(Candida, Tetanus) induced lympho-proliferation”. | “normal NK cell count” | hypo-spadia;  brain:  distinct bilateral small areas sus-picious of focal cortical  hetero-topy | 6,8 |
|  |  | Turkish | m | died at  4½ y |  | hypertelorism | retarded | delay of speech | several bacterial infections  meningo-encephalitis | **IgG very low**  **IgM very low IgA absent** | “autopsy  findings: **absent germinal centers of lymph nodes,**  **thymic cortex atrophy**” |  | hypospadia,  um-bilical hernia | 8 |
| c.833C>G / c.1222T>G | p.S278* / p.C408G | German | m | died at  7 y |  | broad nasal bridge  hypertelorism  flat philtrum | walked at 2 y  muscular hypotonus of lower extremities | retarded | pyelonephritis  recur. otitis pneumonia  gastroenteritis recur. impetigo | **IgG < 2 g/l**  **IgM 0 g/l**  IgA normal | “no T cell deficiency” |  | Hodgkin lymph-oma | 1,2,6,9 |
| c.833C>G / c.1222T>G | p.S278* / p.C408G | German | f | 9 y |  | broad nasal bridge  hypertelorism  flat philtrum | retarded | retarded |  | **“hypogamma-globulinemia”** |  |  |  | 1,2,6,9 |
| c.917delA | p.N306I fs*4 | Turkish | m | 4 y |  | present | retarded | retarded |  | **“agamma-globulinemia”** |  |  |  | 1,6 |
| c.958C>T | p.R320* | Turkish | f | died at 11 y |  | present | retarded | retarded | pseudomonas sepsis | **IgG 4.25 g/l**  **IgM 0 g/l**  **IgA 0 g/l** | “normal T cell count” |  |  | 1,2,6 |
| c.958C>T | p.R320* | Japanese | m | died at 41 y | birth weight 2600g | hypertelorism  micrognathia |  | IQ 47  delay of speech  learning diffi-culties | recur.  pneumonia recur.  sinusitis  JC virus associated leukoence-phalopathy | **B cells 1%**  **CD27+ B cells 0,34% (> 15%)**  **IgG 1,08 g/l**  **(5,7 – 17 g/l)**  **IgM 0,16 g/l**  **(0,33 – 1,9 g/l)**  **IgA 0,29 g/l**  **(1,1 – 4,1 g/l)**  **IgG1 0,5 g/l**  **(3,2 – 7,4 g/l)**  **IgG2 0,34 g/l (2,08 – 7,54 g/l)**  **IgG3 0,381 g/l (0,66 – 8,8 g/l)**  **IgG4 0,16 g/l**  **(0,48 – 1,08 g/l)**  **“no antibodies to vaccines”** | **T cells 99%**  **CD4/CD8 0,32**  **Lymphocyte proliferation:**  **PHA 3150 cpm**  **(20500 – 56800 cpm)** | **NK cell activity 8%**  **(18% – 40%)** |  | 5 |
| c.980_981delGT /  c.787A>T | p.C327W fs*54 / p.K263* | Cape Verdean | f | 16 y |  | epicanthic folds |  | IQ < 70  delay of speech  no reading until 16 y | recur. thrush by candida in first y  recur. ear, nose and throat infections  recur. bacterial  pulmonary infections  bronchiectasis and atelectasis.  lobotomy | B cells  352/ μl  (270 – 860/ μl)  **CD27+ B cells 2% (> 15%)**  IgG 8,15 g/l  (7,7 – 17 g/l)  **IgM 0,18 g/l (0,33 – 1,9 g/l)**  IgA 1,28 g/l  (1,1 – 4,1 g/l)  Antibodies:  tetanus 0,32 IU/ml  **∅ against** **pneumococcus**  **∅ IgM isohaemag-glutinines** | CD3+ T cells  2624/ μl  (1000 – 3900/ μl)  CD3+CD4+ T cells 1120/ μl  (560 – 2700/ μl)  **CD+CD8+**  **1536/ μl**  **(330 – 1400/ μl)**  **CD4/CD8 0,7** |  |  | 5 |
| c.1148G>A | p.C383Y | Japanese | m | died at  7 y | birth weight 2880 g  height 51 cm  HC 32,7 cm  later macro-cephaly  thin habitus  short stature hypo-pituitarism, | macrocephaly, hypertelorism, epcanthal folds  midface flatness  low nasal root, long flat philtrum  thick lips (upper lips < lower lips)  knock-knee irregular toes hypoplastic primary teeth | delay | normal | refractory diarrhea, recur. respir tract infections | **CD27+ B cells 0,34% (> 15%)**  IgG 10,88 g/l (5,7 – 17 g/l)  IgM 0,16 g/l (0,33 – 1,9)  IgA 0,29 g/l  (1,1 – 4,1 g/l)  IgG1 4,57 g/l (3,2 – 7,4 g/l)  **IgG2 0,41 g/l (2,08 – 7,54 g/l)**  IgG3 4,2 g/l (0,66 – 8,8 g/l)  IgG4 0,06 g/l (0,48 – 1,08 g/l)  **“no antibodies upon vaccination”** | CD4/CD8 3  Lymhocyte proliferation:  PHA 23200 cpm  (20500 – 56800) | NK cell activity 5%  (18 % – 40 %) | bilateral hydro-neph-rosis  skin blisters when fever  freckles when sun butterfly erythema | 5 |
| c.1222T>G | p.C408G | Maroccan | m | 8 y | weight P50-75  height P50-75  head circum-ference between mean - +2SD | broad and depressed nasal bridge | walked at 20 months | IQ 65  first words at 12 months  delay of speech |  | CD22+ B cells  360/ μl  (200 – 1300/ μl)  IgG 10,1 g/l  (6,7 – 17,3 g/l)  IgM 0,19 g/l (0,47 – 3,1 g/l)  IgA 1,47 g/l (0,41 – 3,68 g/l)  IgG1 7,05 g/l  (4 – 10,8 g/l)  IgG2 1,3 g/l (0,85 – 4,1 g/l)  IgG3 1,77 g/l (0,13 – 1,47 g/l)  IgG4 0,03  (0,00 – 1,89 g/l) | CD3+ T cells  1846/ μl  (1000 – 3900/ μl)  CD3+CD4+ T cells 854/ μl  (560 – 2700/ μl)  CD+CD8+  728/ μl  (330 – 1400/ μl)  CD4/CD8 1,17 | CD16+ NK cells  104/ μl  (200 –400/ μl) | multiple café-au-lait spots since birth | 3,6 |
| c.1222T>G | p.C408G | German | f | 9 y | birth weight  failure to thrive since 4 y  weight <<P3  height <<P3 | round face  frontal bossing  Hypertelorism  flat philtrum  small chin  retrognathism | no retardation or delay reported | Delay of speech  Selec-tive mutism |  | CD19+ B cells  20/ μl *  (200 – 1600/ μl)  IgG 2,29 g/l  (5 – 13,6 /l)  IgM < 0,1 g/l (0,46 – 1,9 g/l)  IgA 0,33 g/l (0,45 – 1,35 g/l)  IgG1 1,35 g/l  (2,7 – 8,1 g/l)  IgG2 0,24 g/l (0,65 – 2,2 g/l)  IgG3 0,36 g/l (0,16 – 0,96 g/l)  IgG4 < 0,03  (0,01 – 0,9 g/l) ** | CD3+ T cells  6160/ μl *  (700 – 4200/ μl)  CD3+CD4+ T cells 1080/ μl *  (300 – 2000/ μl)  **CD+CD8+**  **4880/ μl** *  (300 – 1800/ μl)  **CD4/CD8 0,22**  **0% CD4+CD45RA+**  **97% CD4+CD45RO+** | CD16+/56+ NK cells  20/ μl *  **NK cell activity 10%**  **(20% – 50%)** | massive hepato-splenomegaly  en-larged kidneys  with granulomatous lympho-cytic infil-trates | this study |
| c.1369C>T | p.R457* | Italian | m | adult | birth weight 2950 g  “Failure to thrive” | epicanthus  hypertelorism  flat nasal bridge  low set ears |  | IQ 61 | bronchiolitis  recur. respiratory tract infections  chronic broncho-pneumonia  bronchiectasis | “normal B cell count”  **IgG 3,8 g/l**  **(5,50 – 16 g/l)**  IgM 1,24 g/l  (0,4 – 2,3 g/l)  IgA 0,2 g/l  (0,4 – 2,3 g/l) | **CD4/CD8 0,65** | “normal NK-cell count” |  | 1,2,10 |

# non-consanguineous family

* Lymphocyte subsets at age 8 2/12

** Immunoglobuline values prior to substitution at 3 5/12 years

1 Greef et al. 2011 2 Hagleitner et al. 2008 3 Cerbone et al. 2012 4 Chouery et al. 2011 5 Nitta et al. 2013

6 Wemaes et al. 2013 7 Brown et al. 1995 8 Kloeckner et al. 2005 9 Schuetz et al. 2007 10 Pezollo et al. 2002
