# Supplementary material for: Combined immunodeficiency develops with age in Immunodeficiency-centromeric instability-facial anomalies syndrome 2 (ICF2)
Source: Orphanet J Rare Dis. 2014 Oct 21;9:116. doi: 10.1186/s13023-014-0116-6 (PMC4230835; doi:10.1186/s13023-014-0116-6)
Supplement: Additional file 2: Table S2 — Immunoglobulin levels and antibody titers. [file 13023_2014_116_MOESM2_ESM.docx]

**Additional file 2: Table S2. Immunoglobulins and antibody titers**

| **Age (years)** | **3.4** | **Normal values**  **according to age** | **8.6** | **9** | **Normal values**  **according to age** | **9.6** | **Normal values**  **according to age** |
| --- | --- | --- | --- | --- | --- | --- | --- |
| **IgA (mg/ dl)** | 33 | 30 - 188 | 1010 | **5150** | **340 - 3500** | **9380** | **530 - 2400** |
| **IgM (mg/ dl)** | < 10 | 43 – 175 | 740 | 630 | 310 - 2080 | 640 | 310 - 1790 |
| **IgG (mg/ dl)** | 229 | 540 – 1340 | 7150 | 7444 | 5720 - 14740 | 9740 | 6980 - 15600 |
| **IgG_1_ (mg/ dl)** | 135 | 270 – 810 |  |  |  |  |  |
| **IgG_2_(mg/ dl)** | 24 | 65 – 220 |  |  |  |  |  |
| **IgG_3_(mg/ dl)** | 36 | 16 – 96 |  |  |  |  |  |
| **IgG_4_(mg/ dl)** | < 0,3 | 1 - 94 |  |  |  |  |  |
| **Antibodies against**  **Tetanus Toxoid - IgG** | not detectable |  |  |  |  |  |  |
| **Antibodies against**  **Pneumococci - IgG** | not detectable |  |  |  |  |  |  |
| **Antibodies against**  **Pneumococci – IgG_2_** | not detectable |  |  |  |  |  |  |
