# Supplementary material for: Combined immunodeficiency develops with age in Immunodeficiency-centromeric instability-facial anomalies syndrome 2 (ICF2)
Source: Orphanet J Rare Dis. 2014 Oct 21;9:116. doi: 10.1186/s13023-014-0116-6 (PMC4230835; doi:10.1186/s13023-014-0116-6)
Supplement: Additional file 3: Table S3 — Blood counts and lymphocyte subpopulations. [file 13023_2014_116_MOESM3_ESM.docx]

**Additional file 3:Table S3. Blood counts and lymphocyte subpopulations**

| **Age (years)** | **2.5** | **2.6** | **3.3** | **4.3** | **Normal (2 – 5 years)** | **5.3** | **5.8** | **7.4** | **8.2** | **Normal (5 – 10 years)** |
| --- | --- | --- | --- | --- | --- | --- | --- | --- | --- | --- |
| **Hemoglobin (g/dl)** |  | **9,9** | **10,4** |  |  | **10,1** | **11,1** | **11,5** | **10,4** |  |
| **Erythrocytes/ pl** |  | **4,85** | **5,53** |  |  | **4,76** | **4,96** | **4,78** | **3,84** |  |
| **MCV (fl)** |  | **63** | **59** |  |  | **67** | **68** | 75 | 80 |  |
| **MCH (pg)** |  | **20,4** | **18,8** |  |  | **21,2** | **22,4** | **24,1** | 27,1 |  |
| **RDW (%)** |  | 15,5 | 18,1 |  |  | 19,4 | 18,5 | 17,6 | 19,5 |  |
| **Reticulocytes (%)** |  |  |  |  |  |  |  |  | 3.1% |  |
| **Reticulocytes/μl** |  |  |  |  |  |  |  |  |  |  |
| **Reticulocyte-Hemoglobin (pg)** |  |  |  |  |  |  |  |  | 24.6 | 28.5 – 34.5 |
| **Thrombocytes/ μl** |  | 328000 | 316000 |  |  | 170000 | **121000** | **67000** | **76000** |  |
| **Leucocytes/ μl** |  | 7700 | 7200 |  |  | 5870 | 6590 | 8010 | 9300 |  |
| **Neutrophilic granulocytes/ μl** |  | 3620 | 2611 |  |  | 1983 | 2155 | 1530 | 2300 |  |
| **Eosinophilic granulocyts/ μl** |  | 20 | 0 |  |  | 0 | 0 | 10 | 10 |  |
| **Basophilic granulocytes/ μl** |  | 0 | 0 |  |  | 0 | 10 | 10 | 20 |  |
| **Monocytes/ μl** |  | 770 | 768 |  |  | 481 | 457 | 510 | 560 |  |
| **Lymphoytes/ μl** | 3053 | 3080 | 4300 |  |  | 3546 | 3918 | 5950 | 6390 | 1500 - 6500 |
| **CD3+/ μl** | 2381 |  | 3838 | 3313 | 900 - 4500 | 3404 | 3840 | 5760 | 6160 | 700 - 4200 |
| **CD3+CD4+/ μl** | 1233 |  | 1333 | 748 | 300 - 2000 | 851 | 823 | 1370 | 1080 | 300 - 2000 |
| **CD3+CD8+/ μl** | 1089 |  | **2452** | **2600** | **300 - 1800** | **2482** | **2978** | **4260** | **4880** | **300 - 1800** |
| **CD4/CD8 ratio** | 1,13 |  | **0,54** | **0,28** | **0,9 – 2,9** | **0,34** | **0,28** | **0,32** | **0,22** | **0,9 – 2,6** |
| **CD3+CD4+CD45RA+ (%)** |  |  | **29** | **12** |  | **4** | **1** |  | **0** |  |
| **CD3+CD4+CD45R0+ (%)** |  |  | **65** | **84** |  | **95** | **98** |  | **97** |  |
| **CD3+αβ+ (%)** |  |  | 98 | 99 |  | 99 | 98 |  | 99 |  |
| **CD3+γδ+ (%)** |  |  | 2 | 1 |  | 1 | 2 |  | 1 |  |
| **CD3-CD16+/CD56+** | 92 |  | 172 | 71 | 100 - 1000 | 165 | 39 | 40 | 20 |  |
| **CD19+** | 488 |  | 258 | 71 | 200 - 2100 | 124 | 39 | 60 | 20 |  |
