# Supplementary material for: Combined immunodeficiency develops with age in Immunodeficiency-centromeric instability-facial anomalies syndrome 2 (ICF2)
Source: Orphanet J Rare Dis. 2014 Oct 21;9:116. doi: 10.1186/s13023-014-0116-6 (PMC4230835; doi:10.1186/s13023-014-0116-6)
Supplement: Additional file 4: Table S4 — Lymphocyte proliferation assays. [file 13023_2014_116_MOESM4_ESM.docx]

**Additional file 4:Table S4. Lymphocyte proliferation assays.**

| **age (years)** | **3.3** | **4.3** | **5.3** | **8.2** |
| --- | --- | --- | --- | --- |
| **medium only control** | 18 | 22 | 21 | 21 |
| **medium only patient** | 20 | 16 | 151 | 23 |
| **PHA control** | 89982 | 84663 | 81593 | 82530 |
| **PHA patient** | **57326** | **38047** | **53923** | **44798** |
| **IL-2 control** | 3966 | 6869 | 9800 | 7126 |
| **IL-2 patient** | 4764 | 2688 | 5422 | 26885 |
| **anti-CD3 control** | 28416 | 35121 | 36458 | 17463 |
| **anti-CD3 patient** | **3264** | **3009** | **5046** | 24601 |
| **PWM control** | 34841 | 29956 | 20142 | 29098 |
| **PWM patient** | 23453 | **5582** | **6699** | **5149** |
| **SAC control** | 299 | 1598 | 2808 | 1259 |
| **SAC patient** | 4588 | **73** | **64** | **100** |
| **tetanus toxoid control** | 3904 | 7310 | 254 | 14076 |
| **tetanus toxoid patient** | **310** | **108** | **258** | **48** |
| **Diphtheria toxoid control** | 221 | 2544 | 21 | 8927 |
| **Diphtheria toxoid patient** | 772 | 551 | 609 | 56 |
| **PPD control** | 11621 | 2734 | 140 | 72 |
| **PPD patient** | 111 | 140 | 200 | 22 |
| **Candida control** | 1385 | 5760 | 1598 | 315 |
| **Candida patient** | 294 | 209 | 657 | 71 |

values in cpm, counts per minute
