# Supplementary material for: Combined immunodeficiency develops with age in Immunodeficiency-centromeric instability-facial anomalies syndrome 2 (ICF2)
Source: Orphanet J Rare Dis. 2014 Oct 21;9:116. doi: 10.1186/s13023-014-0116-6 (PMC4230835; doi:10.1186/s13023-014-0116-6)
Supplement: Additional file 5: Table S5 — NK-cell cytotoxicity assay. Percentages of lysed K562 cells at different effector:target ratios with and without supplementation with IL-2 at the age of 4 years are shown. [file 13023_2014_116_MOESM5_ESM.docx]

**Additional file 5:Table S5. NK-cytotoxicity assay depicting percentage of lysed K562 cells at different effector:target ratios with and without supplementation with IL-2 at the age of 4 years.**

| **Effector:Target ratio** | **patient** | **control** |
| --- | --- | --- |
| **50:1** | 10 | 36 |
| **50:1 + IL-2** | 31 |  |
| **25:1** | 9 | 24 |
| **25:1 + IL-2** | 16 |  |
