# Supplementary figures and images for: Combined immunodeficiency develops with age in Immunodeficiency-centromeric instability-facial anomalies syndrome 2 (ICF2)
Source: Orphanet J Rare Dis. 2014 Oct 21;9:116. doi: 10.1186/s13023-014-0116-6 (PMC4230835; doi:10.1186/s13023-014-0116-6)

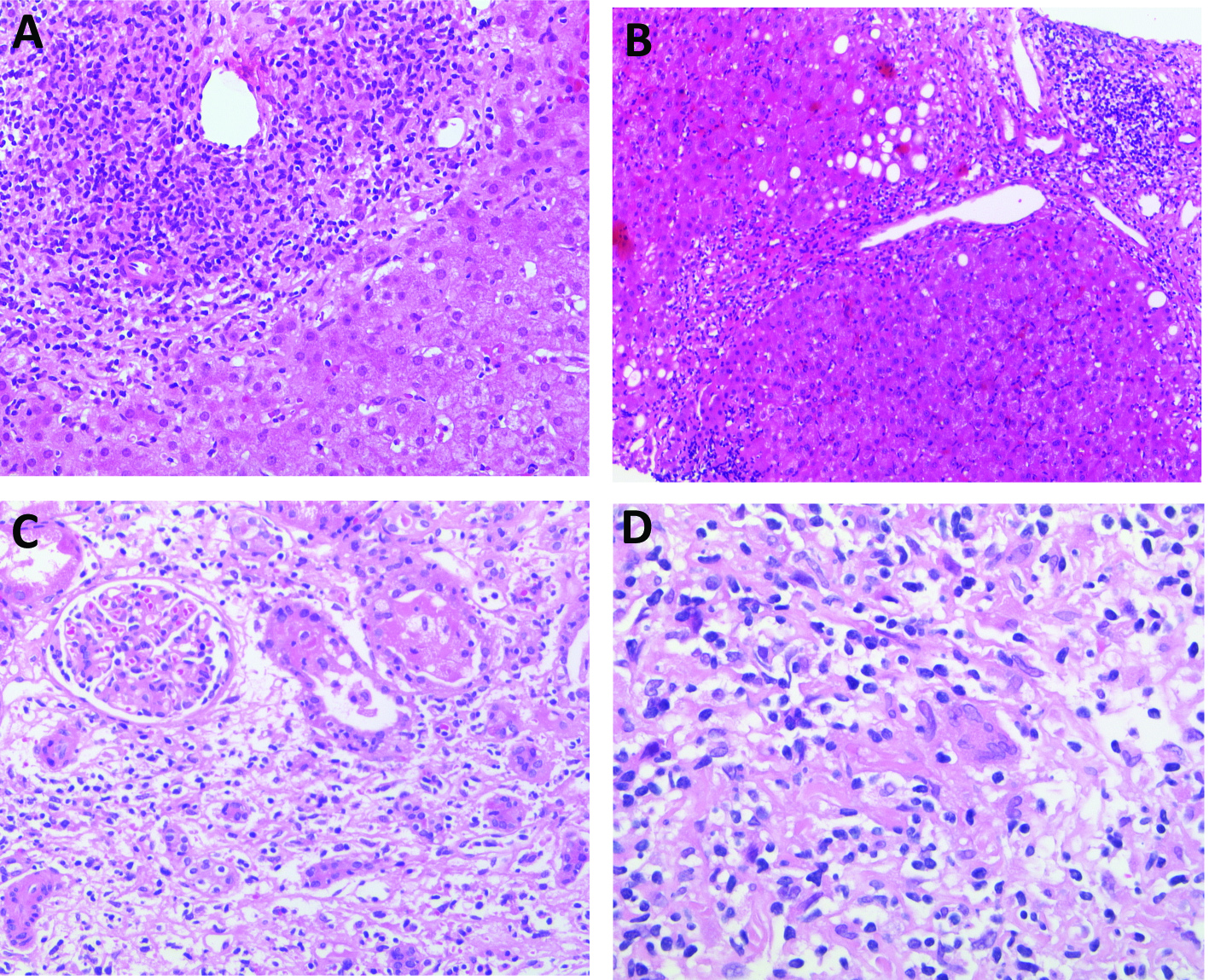

Supplement: Additional file 6: Figure S1 — Histological biopsy results. (A) Liver biopsy at the age of 4,5 years. Portal lymphocytic infiltrates and interface hepatitis. (B) Liver biopsy at the age of 8,5 years. Porto-portal bridging. (C) Kidney biopsy at the age of 4,5 years. Normal glomerula. (D) Kidney biopsy at the age of 4,5 years showed multifocal inflammatory infiltrates in cortex and medulla of the tubulo-interstitium and a multinuclear giant cell. [file 13023_2014_116_MOESM6_ESM.jpeg]

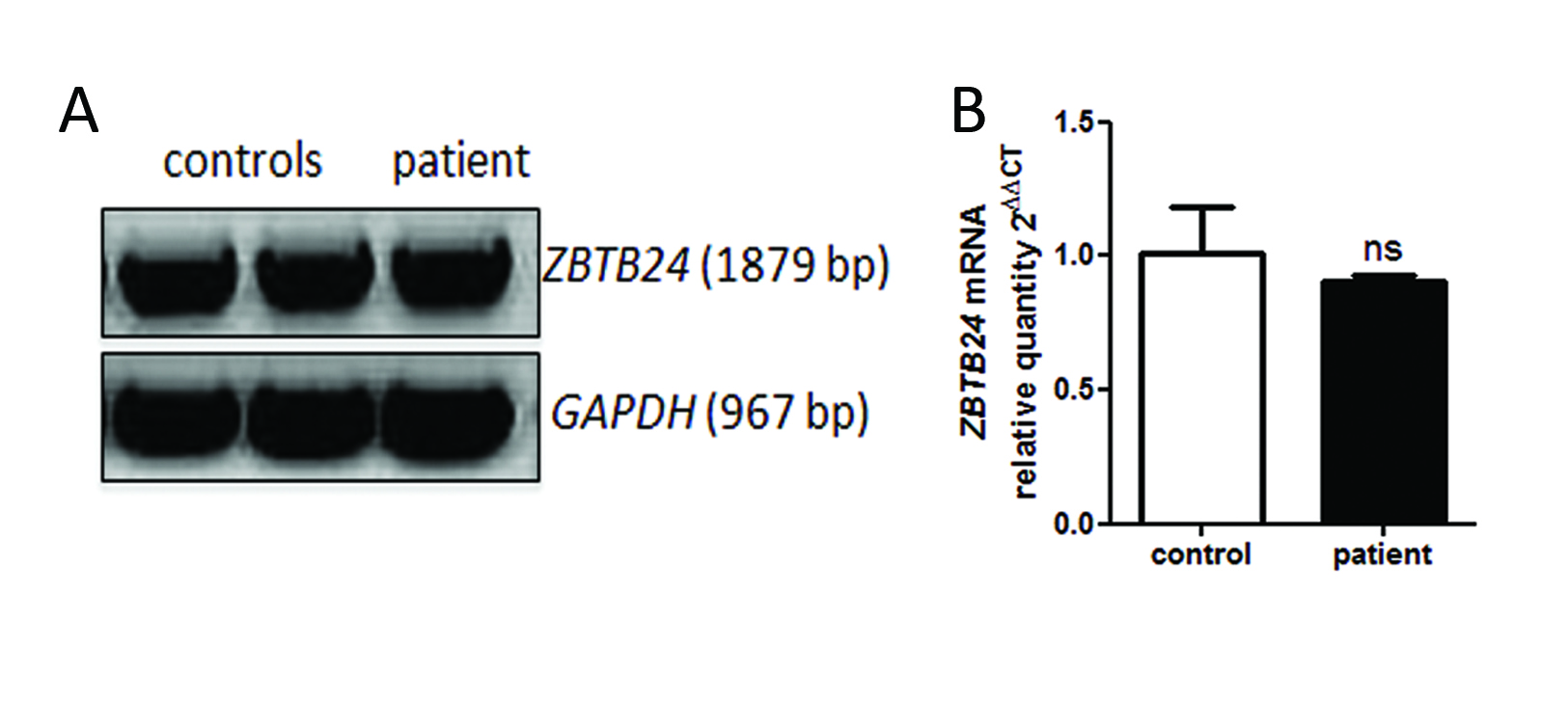

Supplement: Additional file 8: Figure S2 — ZBT24 mRNA levels in controls and fibroblasts. (A) RT-PCR of ZBTB24 in fibroblasts of controls and the patient. (B) Quantitative RT-PCR of ZBTB24 in fibroblasts of a control cell line and the patient. [file 13023_2014_116_MOESM8_ESM.jpeg]

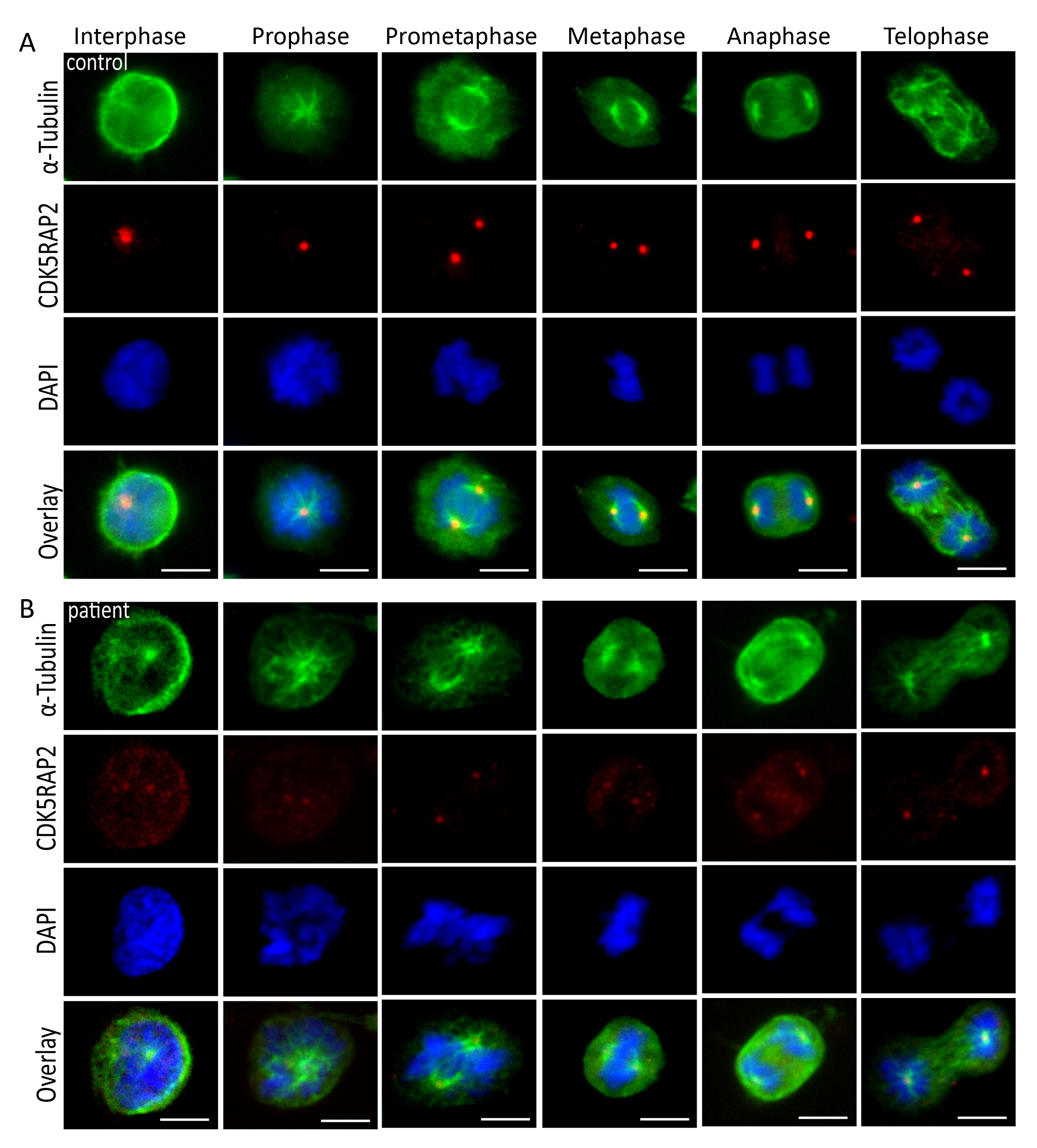

Supplement: Additional file 9: Figure S3 — Mitotic spindles defect in ZBTB24 mutant patient cells. Subcellular localization of the spindle marker α-tubulin (green) and the centrosome marker CDK5RAP2 (red) of immortalized lymphocytes of (A) control and (B) ICF2 patient throughout the cell cycle. DNA was stained with DAPI (blue). Patient cells have abnormal spindle formation with an increase of slightly broader, unfocused microtubules poles. The fluorescence signals of the centrosomal marker CDK5RAP2 are strongly reduced in patient cells when compared to control cells. Immunofluorescence, scale bar 5 μm. [file 13023_2014_116_MOESM9_ESM.jpeg]

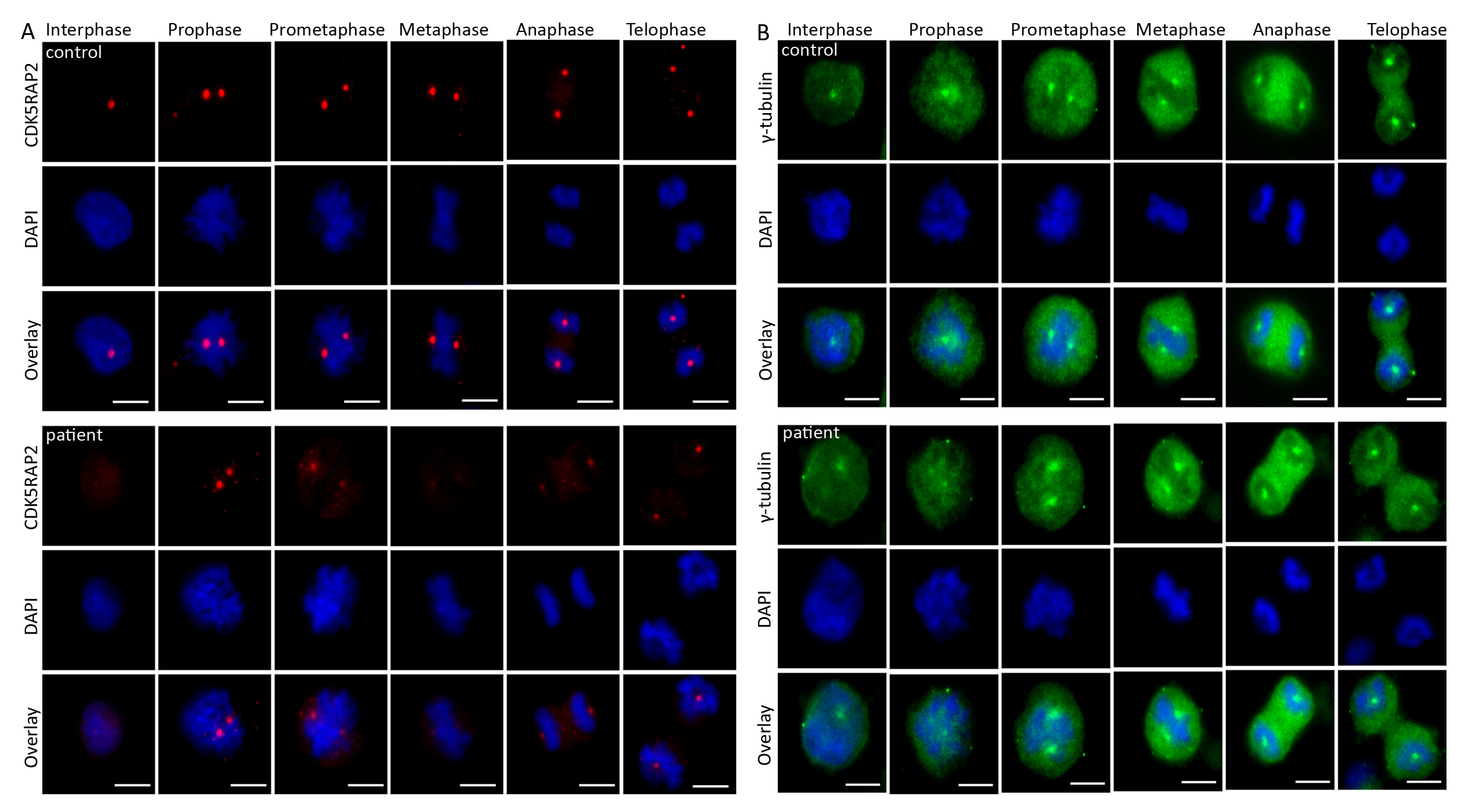

Supplement: Additional file 10: Figure S4 — Abnormal CDK5RAP2 and normal γ-tubulin staining of centrosomes in ZBTB24 mutant patient cells. (A) Subcellular localization of the centrosome marker CDK5RAP2 (red) in the metaphase of immortalized lymphocytes of control and ICF2 patient. DNA was stained with DAPI (blue). Centrosomal CDK5RAP2 is strongly reduced in ZBTB24 mutant lymphocytes when compared to controls. (B) Subcellular localization of the centrosome marker γ-tubulin (green) in the metaphase of immortalized lymphocytes of control and ICF2 patient. DNA was stained with DAPI (blue). No strong difference between the γ-tubulin immunostaining of control and ICF2 patient Immunofluorescence, scale bar 5 μm. [file 13023_2014_116_MOESM10_ESM.jpeg]

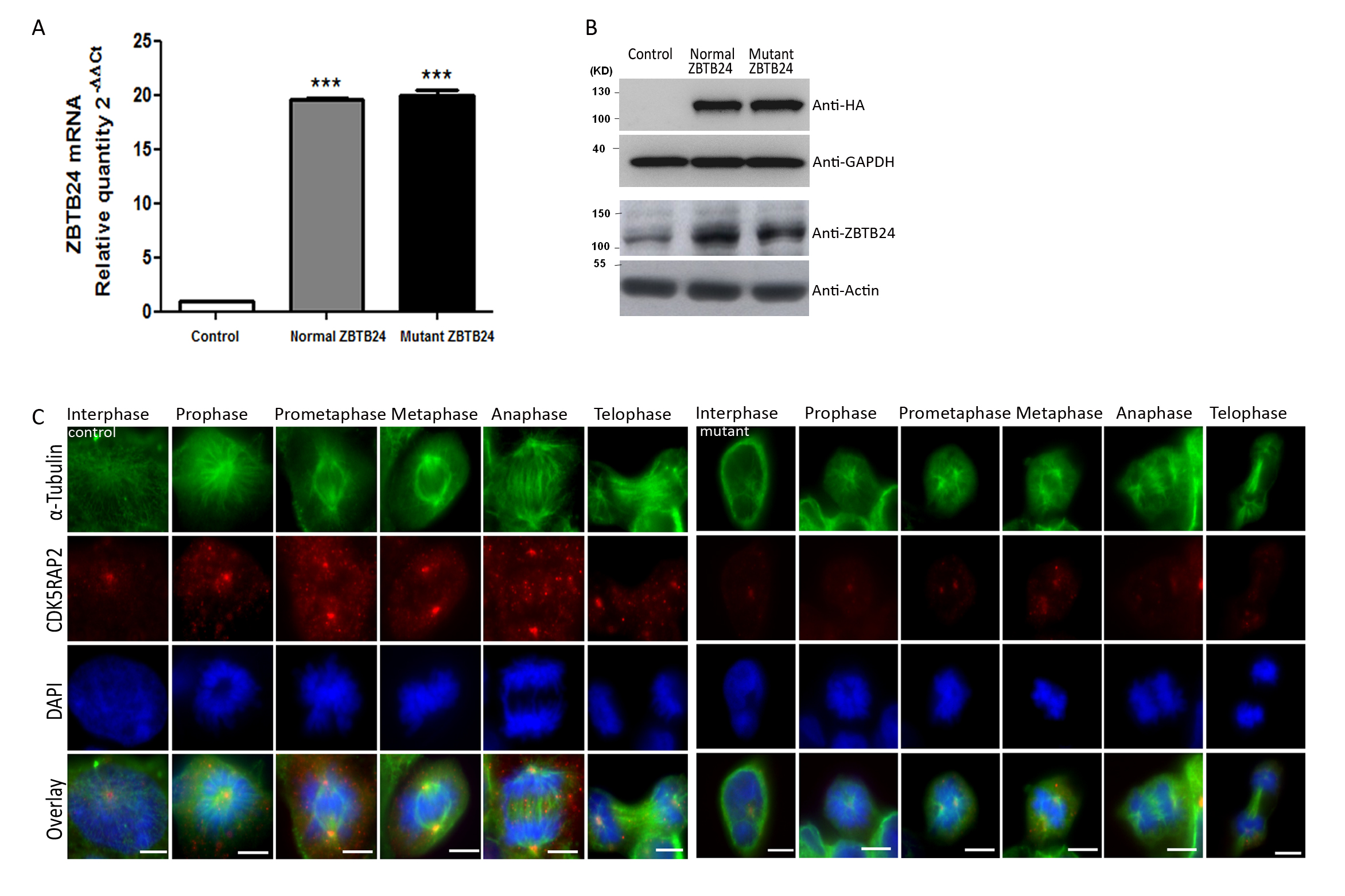

Supplement: Additional file 11: Figure S5 — Mimicking ICF2 in HEK cells. (A) ZBTB24 mRNA levels and in mock-transfected HEK cells (“control”), HEK cells transfected with HA-tagged normal ZBTB24 or mutant c.1222 T > G ZBTB24, assessed by qPCR. (B) Western-Blot of ZBTB24 of protein extracts from similarly transfected HEK cells using antibodies directed against HA-tag and against ZBTB24 (reference proteins GAPDH and actin). (C) Subcellular localization of the centrosome marker γ-tubulin (green) and the centrosome marker CDK5RAP2 (red) throughout the cell cycle in mock-transfected HEK cells (“control”) and HEK cells transfected with mutant c.1222 T > G ZBTB24. [file 13023_2014_116_MOESM11_ESM.jpeg]
