# Supplementary material for: Combined immunodeficiency develops with age in Immunodeficiency-centromeric instability-facial anomalies syndrome 2 (ICF2)
Source: Orphanet J Rare Dis. 2014 Oct 21;9:116. doi: 10.1186/s13023-014-0116-6 (PMC4230835; doi:10.1186/s13023-014-0116-6)
Supplement: Additional file 7: Table S6 — Pathogens excluded to cause hepatitis. [file 13023_2014_116_MOESM7_ESM.docx]

**Additional file 7:Table S6. Pathogens excluded to cause hepatitis.**

| **Pathogens** | **Method** | **Specimen** |
| --- | --- | --- |
| *Herpesviridae* |  |  |
| Human Herpes Simplex Virus 1 (HSV1) | PCR | Blood & liver biopsy |
| Human Herpes Simplex Virus 2 (HSV2) | PCR | Blood & liver biopsy |
| Epstein Barr virus (EBV) | PCR | Blood & liver biopsy |
| Cytomegalovirus (CMV) | PCR | Blood & liver biopsy |
| Human Herpesvirus 6 (HHV6) | PCR | Blood & liver biopsy |
| Human Herpresvirus 7 (HHV7) | PCR | Blood & liver biopsy |
| *Hepatisisvirdae* |  |  |
| Hepatitis Virus A | PCR | Blood & liver biopsy |
| Hepatitis Virus B | PCR | Blood & liver biopsy |
| Hepatitis Virus C | PCR | Blood & liver biopsy |
| Hepatitis Virus D | PCR | Blood & liver biopsy |
| Hepatitis Virus E | PCR | Blood & liver biopsy |
| Adenovirus | PCR | Blood & liver biopsy |
| Enteroviridae | PCR | Blood & liver biopsy |
| Parvoviurs B19 | PCR | Blood & liver biopsy |
| Hantavirus | PCR | Blood & liver biopsy |
| BK-Virus | PCR | Blood & liver biopsy |
| *Mycobacteriae* | Staining  PCR  Culture | liver biopsy |
