# Supplementary material for: Combined immunodeficiency develops with age in Immunodeficiency-centromeric instability-facial anomalies syndrome 2 (ICF2)
Source: Orphanet J Rare Dis. 2014 Oct 21;9:116. doi: 10.1186/s13023-014-0116-6 (PMC4230835; doi:10.1186/s13023-014-0116-6)
Supplement: Additional file 12 — Materials and Methods. [file 13023_2014_116_MOESM12_ESM.docx]

**Materials and Methods**

Patients. Informed consent was obtained from the parents of the patient for the molecular genetic analysis, the publication of clinical data, radiological imaging data, and studies on lymphoblastoid cell lines (LCLs) as well as primary and immortalized fibroblasts. DNA was extracted from EDTA blood samples using standard procedures. Samples from the patient and her parents were used in this study with approval from the local ethics committees of the Charité (approval nos. EA1/212/08 and EA2/163/12). Blood samples were drawn in parallel to those necessary for routine blood tests.

**Array-CGH analysis.** For array-CGH (comparative genomic hybridization), genomic DNA was isolated from peripheral blood lymphocytes according to standard procedures, labeled using the Genomic DNA Enzymatic Labeling Kit (Agilent, Santa Clara, USA), and hybridized on a Microarray Kit 244A chip (Agilent).

Epstein-Barr virus-transformed lymphocyte and fibroblast culture. LCLs were established according to the protocol published by Neitzel et al. 1986 [[1](#_ENREF_1)]. Non-adherent LCLs were cultured in RPMI 1640 with L-Glutamine (Invitrogen, Darmstadt, Germany) supplemented with 20% v/v fetal bovine sSubject:erum (Biochrom) and 1% v/v penicillin-streptomycin (Sigma-Aldrich, Taufkirchen, Germany). Fibroblasts from our index patient and age-matched control were established according to a standard protocol and cultured in DMEM with 4.5 g/l D-glucose and pyruvate (Invitrogen, Darmstadt, Germany) supplemented with 15% fetal bovine serum and 1% penicillin-streptomycin [[2](#_ENREF_2)]. For this study, we used LCLs from the index patient and from one control.

**Lymphocyte subset and TCRVβ repertoire analysis.** Lymphocyte subsets were stained with fluorescence-labeled monoclonal antibodies against CD14 (RMO52), CD56 (N901), CD16 (3G8), CD4 (SFCI12T4D11), CD19 (J3-119), CD8 (SFCI21Thy2D3), CD3 (UCH-T1), CD45 (J33), CD45R0 (UCHL1), TCRαβ (IP26A), T-cell receptor (TCR) γδ (IMMU510), and CD45RA (2H4LDH11LDB9), IgD (IADB6), HLA-DR (Immu-357), CD24 (ALB9), CD27 (1A4CD27), IgM (UHB), and CD38 (LS198-4-3) (antibodies all from Beckman Coulter, Brea, California, USA; dilutions available upon request) for 15 min at room temperature. Cells were measured and analyzed after erythrocyte lysis (Versa-Lyse, Beckman Coulter) on a NAVIOS-FACS (fluorescence-activated cell sorting; Beckman Coulter). The TCRVß repertoire was analyzed by flow cytometry using the Beckman Coulter TCRVß repertoire panel (antibodies and conditions available upon request). Data were analyzed using Navios software 1.2 (Beckman Coulter).

**NK-cytotoxicity assessment.** Natural killer (NK)-cell-function was measured by K562-cell lysis with the NK-cell-function-assay kit (Glycotope, Berlin, Germany) according to the manufacturer’s guidelines. Fluorescent membrane stained K562-target cells were incubated with Ficoll-Paque purified peripheral blood mononuclear cells (PBMC´s) and the percentage of target cells killed by effector NK cells was determined. For this, effector and target cells were incubated and subsequently a red fluorescent DNA dye was added to label the target cells with compromised plasma membranes, permeabilized by NK activity. Four cell populations could thereby be distinguished (live target cells, dead target cells, live effector cells, and dead effector cells), and the actual ratio between effector and target cells could be determined. Cells were measured and analyzed on a FACSCANTO (Becton Dicinson Biosciences), and data were analyzed using FACSDiva-Software (Becton Dicinson Biosciences).

**Lymphocyte proliferation assay.** Lymphocyte proliferative responses were assayed according to standard protocols in Ficoll-separated peripheral blood mononuclear cells (PBMCs) stimulated with phytohemagglutinin (PHA, Sigma-Aldrich, L1668), plate-bound anti-CD3 (3, 10, and 30 µg/ml; clone HIT3a, Becton Dicinson), Pokeweed-Mitogen (1, 5, and 15 µg/ml, PWM, Sigma-Aldrich, L-9379), IL-2 (20 ng/ml, #200-02, Peprotech, Rocky Hill/NJ, USA), and SAC (Staphylococcus aureus Cowan-1)/Pansorbin® cells (1:1000, 1:10000, Merck, Darmstadt, Germany) for 72 hours and with Tetanus-Toxoid RT 50 (40, 20, and 10 Lf/ml, Staten Serum Inst., Copenhagen, Denmark), Candida albicans antigen (0.025, 0.25, and 2.5 µg/ml; CAN001-P; Biologo Kronshagen, Germany) and Diphtheria-toxoid (20 LF/ml, Chiron-Behring) for 120 hours. After pulsing with 1 μCi [^3^H]-thymidine per well during the last 12 hours incorporated [^3^H]-thymidine was measured on a β-scintillation counter (Microbeta 1450 Trilux, Perkin Elmer). Proliferation was analyzed in comparison to a healthy control donor.

**Exome sequencing and bioinformatic methods.** All three family members were subjected to exome sequencing. Genomic DNA was isolated from blood samples using standard methods. Five micrograms of genomic DNA were enriched using the Agilent Human All Exon V3 kit (Agilent Technologies, Santa Clara, CA, USA) following the manufacturer's protocol. Whole-exome libraries were sequenced on an Illumina HiSeq 2000 system for 1x101 cycles following the manufacturer's instructions (Illumina, San Diego, CA, USA). In total, we obtained 92.3-111.4 million single-end 101 bp reads per sample, of which 98.4-98.5% could be mapped onto the human genome. After removing duplicated reads, which were possibly derived from PCR artifacts, 31.2-34.0 million unique reads were mapped to the targeted protein coding regions, resulting in an average of 62.6-68.3 fold-coverage within the targeted coding region. Using the GATK, we detected 19,699-19,836 SNPs and INDELs in the exome of each family member, of which 98.3-98.4% were known variants deposited in dbSNP 135. Given the pedigree, we searched for autosomal-dominant, autosomal-recessive homozygous or compound heterozygous inheritance patterns. All raw sequencing reads were mapped onto UCSC hg19 using BWA 0.5.9-r16, mappings were converted into BAM file format using samtools 0.1.18. Initial mappings were post-processed using GATK 1.6 following their 'best practices V3'. In brief, reads were realigned around sites of known INDELs. Then, likely PCR duplicates were detected using Picard 1.48. Finally, raw base quality scores were empirically recalibrated. Single Nucleotide Polymorphisms (SNPs) and INDELs were identified using the UnifiedGenotyper from GATK. Variants were classified as novel or known variants according to Single Nucleotide Polymorphism Database dbSNP 135. Functional consequences of each variant were annotated using genetic variant annotation and effect prediction toolbox snpEff 2.0.5d20 for UCSC hg19 RefSeq genes and ENSEMBL 65 human gene models. The potential deleterious effect was evaluated using PolyPhen 2 (Polymorphism Phenotyping v2), SIFT (Scale-invariant feature transform), PhyloP, MutationTaster, GERP++ (Genomic Evolutionary Rate Profiling), LRT (Likelihood Ratio Test), and OMIM (Online Mendelian Inheritance in Man), if available. Variants were filtered for autosomal-dominant, autosomal-recessive, and compound heterozygous inheritance patterns.

Sanger sequencing. The missense mutations in the *ZBTB24* gene identified through exome sequencing were verified through Sanger sequencing using standard techniques. The database sequence NM_014797.2 for the *ZBTB24* gene was used as reference, and the following primers for sequencing: *ZBTB24*-F 5’-CCCAAAGACAGTGCTGACAAC-3’, *ZBTB24-*R 5’-GAGGCAGT GAGTTGAGGTGC-3’.

**RNA extraction and RT-PCR.** Total RNA from simian virus 40 (SV40)-transformed fibroblasts and from human embryonic kidney (HEK) 293 cells was extracted using the RNeasy Mini Kit (Qiagen). RNA concentration and purity was evaluated by spectrophotometric measurement, and RNA integrity was verified by clearly visible 28S and 18S ribosomal RNA bands. cDNA was prepared by reverse transcription of 1 µg of RNA using the ThermoScript® RT-PCR System (Invitrogen), using a combination of oligo(dT)_20_ and random hexamer primers. *ZBTB24* transcripts were detected by PCR using primers *ZBTB24-*F 5’-TTCTTCTGTCGCCGGCTTCAG-3’, *ZBTB24*-R 5’-CATGCTTTAATAACCA CTGTCCTGC-3’ *GAPDH*-F 5´-CGCTCTCTGCTCCTCCTGTTCG-3´, and *GAPDH*-R 5´-GCGTCAAAGGTGGAGGAGTGGG- 3´.

**Quantitative PCR for *ZBTB24* mRNA level.** For quantitative PCR (qPCR), total RNA was extracted from HEK293 cells to synthesize single-strand cDNA. Primers *ZBTB24*-F 5’- AGAGCCATTACCGAGTTCATACAG -3’ and *ZBTB24*-R 5’- AAGATTTGCCACAGATTTCACAAG -3’ were used to detect *ZBTB24* transcripts. *GAPDH* gene was amplified for normalization by using primer pair *GAPDH*-F 5´-AGCCACATCGCTCAGACAC-3´ and *GAPDH*-R 5´-GCCCAATACGACCAAA TCC-3´. qPCR was performed on the LightCycler® 480 System Cycler (Roche, Basel, Switzerland) with the [SYBR® Green PCR Master Mix](http://docs.appliedbiosystems.com/pebiodocs/04310251.pdf) (Roche) and the following amplification condition: an initial denaturation at 95 °C for 3 min, followed by 42 cycles of 95 °C for 15 s, 60 °C for 15 s, and 72 °C for 15 s, and a final extension cycle at 72 °C for 5 min. Each sample was analyzed in triplicates.

**Protein extraction and Western blot.** Protein extracts for Western blots were isolated from fibroblasts and LCLS by homogenization in radio-immunoprecipitation assay (RIPA) buffer containing 1 mM phenylmethylsulfonyl fluoride (PMSF; Sigma-Aldrich) and 1 protease inhibitor cocktail tablet per 10 ml RIPA buffer (Complete Mini; Roche Diagnostics, Mannheim, Germany), 15 min incubation on ice, followed by ultrasonication treatment and centrifugation for 20 min at 16,000 g and 4 °C. Protein concentrations were determined using a bicinchoninic acid-based assay, according to the instructions of the manufacturer (BCA Protein Assay Kit; Pierce Biotechnology, Rockford, IL, USA). Protein extracts (35 µg per sample) were denaturated in Laemmli sample loading buffer at 95 °C for 5 min, separated by sodium dodecyl sulphate polyacrylamide gel electrophoresis (SDS-PAGE) and electrophoretically transferred onto nitrocellulose membrane (Bio-Rad, Munich, Germany) using a Bio-Rad wet transfer system. The membranes were incubated for 1 h at room temperature in blocking buffer (Tris-buffered saline with Tween (TBS-T) 1x with 5% bovine serum albumin (BSA, Sigma-Aldrich), followed by overnight incubation at 4 °C with rabbit anti-ZBTB24 (1:250, AP54609PU-N, Acris), mouse anti-actin (1:10,000, MAB1501, Millipore), rabbit anti-HA and mouse anti-GAPDH (both Sigma-Aldrich) antibodies in the blocking solution. After incubation with the corresponding secondary antibodies donkey anti-rabbit (1:4,000; Amersham Biosciences, Freiburg, Germany) and goat anti-mouse (1:10,000; Dako, Hamburg, Germany), the immunoreactive proteins were visualized using a technique based on a chemiluminescent reaction. The gel pictures were obtained using photographic films (Amersham Hyperfilm enhanced chemiluminescence (ECL, GE Healthcare, UK)). Western blot experiments were run in triplicates.

**Neutrophil phenotype analysis.** Neutrophils were isolated from heparinized peripheral blood as described previously [[3](#_ENREF_3)]. Briefly, whole blood was separated by spinning on a Histopaque 1119 cushion (Sigma-Aldrich, St. Louis, USA). The resulting neutrophil-rich fraction was washed and disposed on a discontinuous Percoll gradient (85–65% in PBS 1x). Following centrifugation at 800 g for 20 min at room temperature, bands were collected from the 80, 75, and 70% layers, pooled, and washed with phosphate buffered saline (PBS) 1x. Cells were spun onto slides using a Shandon Cytospin 3 Centrifuge (Thermo Scientific, Rockford, United States) and stained with Wright-Giemsa dye (Sigma-Aldrich). For nuclear lobulation analyses, 200 cells from 2 different blood draws were evaluated.

Immunocytology. For fixation, LCLs were plated on poly-L-lysine (Sigma-Aldrich) coated coverslips, cultured for 60 min in standard conditions, and incubated in 37 °C paraformaldehyde (PFA) 4% for 10 min prior to rinsing with PBS 1x. Coverslips were further incubated at room temperature in staining buffer (0,2% gelatin, 0,25% Triton X-100 in PBS 1x) for 20 min and subsequently in 10% donkey normal serum (DNS) in staining buffer for 30 min for blocking. Coverslips were incubated overnight at 4 °C with primary antibodies in the staining buffer containing 10% DNS followed by an incubation with the corresponding secondary antibodies for 2 h at room temperature. Nuclei were labeled with 4’,6-diamidino-2-phenylindole (DAPI, 1:1,000, Sigma-Aldrich). Immocytological analysis was also performed with HEK cells. Fluorescently labeled cells were analyzed and imaged by a fluorescent Olympus BX51 microscope with the software Magnafire 2.1B (Olympus, Hamburg, Germany), and all images were processed using Adobe Photoshop and ImageJ. Primary antibodies were as follows: mouse anti-γ-tubulin (T6557, Sigma-Aldrich, 1:500), mouse anti-alpha-tubulin (T9026, Sigma-Aldrich; 1:1500), rabbit anti-Cyclin-dependent kinase 5 regulatory subunit-associated protein 2 (CDK5RAP2, HPA035820, Sigma-Aldrich, 1:200).

**Quantification of cell viability, apoptosis, and proliferation.** Control and index patient fibroblasts were plated at a density of 10^3^ cells per 100 µl and well in 96-well plates for experiments performed to quantify cell viability, proliferation, and apoptosis. All measurements were taken using Synergy 2 Multi-Mode Microplate Reader (BioTek Instruments, Winooski, VT, USA), and the Gen5^TM^ software version 1.02.8 (BioTek Instruments), and data were analyzed using GraphPad Prism. Cell viability was quantified 12, 36, 60, 84, and 108 hours following plating of cells using the fluorimetric CellTiter-Blue Cell Viability Assay® (Promega, Madison, WI) according to the instructions of the manufacturer. The fluorescence values were measured at 560/590 nm. To analyze the proliferation rate of fibroblasts, the 5-bromo-2-deoxyuridine (BrdU) Cell Proliferation ELISA® (Roche, Mannheim, Germany) was performed according to the manufacturer‘s instructions. Here, the absorbance values were measured at A370 nm 36 hours after plating. The apoptosis of fibroblasts was assessed using the ApoONE Homogeneous Caspase 3/7 Assay® (Promega, Madison, WI). Measurements were taken at five different time points (12, 36, 60, 84, and 108 hours) at 485/527 nm. Cell viability was also quantified using HEK cell cultures.

**Cell cycle analysis.** Cell cycle analysis was performed using the 5-bromo-2’-deoxyuridine (BrdU)-Hoechst 33258 method [[4](#_ENREF_4)]. The principle of that assay is based on the incorporation of the halogenated base analog during DNA replication. The assay makes use of the fact that BrdU-substituted chromatin quenches the fluorescence of the dye Hoechst 33258. This method differentiates not only between cycling and noncycling cells in a given culture but also recognizes the distribution of the cycling cells in as many as four consecutive cycles. For flow cytometry, mononuclear from heparinized blood samples were Ficoll-isolated. Cultures were set up in RPMI 1640 medium with GlutaMAX (Gibco Life Technologies), supplemented with 15% FBS (PAN Biotech). Duplicate cultures were either left untreated or irradiated with 1.5 Gy from a linear accelerator or continuously exposed to 10 ng/ml mitomycin C (Medac). To all of the cultures, BrdU (Sigma-Aldrich) was added at a final concentration of 10^-4^ M (25). Lymphocyte growth activation was induced by phythemagglutinin (PHA, HA16, Remel Europe, Dartford, UK). The cells were harvested after 72 h. Following staining with Hoechst 33258 (1.2 μg/ml; Molecular Probes) for a minimum of 15 min in buffer containing 154 mM sodium chloride (NaCl), 0.1 M TRIS pH 7.4, 1 mM calcium chloride (CaCl_2_), 0.5 mM magnesium chloride (MgCl_2_), 0.2% BSA and 0.1% nonyl phenoxypolyethoxylethanol (NP40) in distilled water in the dark, ethidium bromide (EB, 1.5 μg/ml, Molecular Probes) was added for another minimum of 15 min. Bivariate flow histograms were recorded on a triple-laser equipped LSRII flow cytometer (Becton Dickinson) using UV excitation of Hoechst 33258 and 488-nm excitation of EB. The resulting cell cycle distributions reflecting cellular DNA content were quantified using the MPLUS AV software package (Phoenix Flow Systems, San Diego, CA, USA).

**Metaphase preparation and analysis of chromosome condensation.** For chromosome analysis peripheral blood lymphocytes from the index patient and her parents were karyotyped. Whole-blood cultures were prepared using RPMI 1640 medium with GlutaMAX (Gibco Life Technologies) supplemented with 15% FBS (PAN Biotech). Lymphocytes were stimulated with PHA (Remel Europe). Some of the cultures were exposed to 5-azacytidine at a concentration of 1x10^-5^ M for the last 7 h. The cultures were terminated after 72 h following addition of 8 μl/ml Colcemid solution (10 μg/ml; PAA) for the final 45 min. Metaphase preparations were made by hypotonic incubation of the cell pellets (0.075 M KCl for 10 min at 37 °C) and fixation of the nuclei in ice-cold methanol/concentrated acetic acid 3:1. Slides were stained with 5% Giemsa solution for 5 min without applying banding techniques. Alternatively, chromosomes were GTG-banded using standard techniques. A total of 500 metaphases were scored and the proportion of chromosomes with undercondensation of constitutive heterochromatin was determined.

***ZBTB24* siRNA.** *ZBTB24* siRNA knockdown experiments were conducted using vectors from Ambion, si19038 targeting human *ZBTB24* CDS. The ZBTB24 knockdown efficiency was tested by checking the mRNA levels in the transfected *HEK* cells by quantitative PCR, which indicates a reduction of *ZBTB24* mRNA levels in the knockdown system.

**References**

1. Neitzel H: **A routine method for the establishment of permanent growing lymphoblastoid cell lines.** *Hum Genet* 1986, **73:**320-326.

2. Issa L, Mueller K, Seufert K, Kraemer N, Rosenkotter H, Ninnemann O, Buob M, Kaindl AM, Morris-Rosendahl DJ: **Clinical and cellular features in patients with primary autosomal recessive microcephaly and a novel CDK5RAP2 mutation.** *Orphanet J Rare Dis* 2013, **8:**59.

3. Aga E, Katschinski DM, van Zandbergen G, Laufs H, Hansen B, Muller K, Solbach W, Laskay T: **Inhibition of the spontaneous apoptosis of neutrophil granulocytes by the intracellular parasite Leishmania major.** *J Immunol* 2002, **169:**898-905.

4. Rabinovitch PS, Kubbies M, Chen YC, Schindler D, Hoehn H: **BrdU-Hoechst flow cytometry: a unique tool for quantitative cell cycle analysis.** *Exp Cell Res* 1988, **174:**309-318.
